# Evaluation of an Education and Training Program to Prevent and Manage Patients’ Violence in a Mental Health Setting: A Pretest-Posttest Intervention Study

**DOI:** 10.3390/healthcare4030049

**Published:** 2016-08-01

**Authors:** Stéphane Guay, Jane Goncalves, Richard Boyer

**Affiliations:** 1 Trauma Studies Centre, Centre de Recherche—Institut Universitaire en Santé Mentale de Montréal, 7401 Hochelaga, Montreal, QC H1N 3M5, Canada; goncalves.jane@ymail.com (J.G.); richard.boyer@umontreal.ca (R.B.); 2 School of Criminology, University of Montreal, Pavillon Lionel-Groulx, 3150 Jean-Brillant, Montreal, QC H3T 1N8, Canada

**Keywords:** aggression, psychiatric hospital, psychological distress, confidence in coping, education and training program, violence

## Abstract

Workplace violence can lead to serious consequences for victims, organizations, and society. Most workplace violence prevention programs aim to train staff to better recognize and safely manage at-risk situations. The *Omega* education and training program was developed in Canada in 1999, and has since been used to teach healthcare and mental health workers the skills needed to effectively intervene in situations of aggression. The present study was designed to assess the impact of *Omega* on employee psychological distress, confidence in coping, and perceived exposure to violence. This program was offered to 105 employees in a psychiatric hospital in Montreal, Canada. Eighty-nine of them accepted to participate. Questionnaires were completed before the training, after a short period of time (M = 109 days) and at follow-up (M = 441 days). Repeated-measures ANOVAs and Cohen’s *d* effect sizes were calculated. Results demonstrated statistically significant improvements in short-term and follow-up posttest scores of psychological distress, confidence in coping, and in levels of exposure to violence. This study is one of very few to demonstrate the positive impact of this training program. Further research is needed to understand how to improve the effectiveness of the program, especially among participants resistant to change.

## 1. Introduction

According to the World Health Organization, workplace violence is “the intentional use of physical force or power, threatened or actual, against oneself, another person, or against a group or community, that either results in or has a high likelihood of resulting in injury, death, psychological harm, maldevelopment or deprivation” [1]. Workplace violence, and particularly client-initiated violence, is a serious problem for mental health workers, as they represent one of the most at-risk populations [2]. Rates of patient aggression in psychiatric settings range from 0.07–0.25 aggressive incidents per bed per year [3]. A literature review from Piquero, Piquero, Craig, and Clipper [4] showed that between 14% and 61% of mental health workers had been victimized by violent acts, mainly due to aggressive patient behaviours [5]. However, the study-specific variations regarding the measure of prevalence (i.e., prevalence period, survey designs) render efforts to establish largely based prevalence rates challenging [6].

The consequences of violent acts are numerous, whether they be on victims (and on their relationship with patients), on organizations, or on society. More precisely, a systematic literature review of the impact of workplace violence in healthcare settings conducted by Lanctôt and Guay [7]**,** identified 68 studies that address different types of potential consequences. Among these, 47 studies identified several psychological consequences for workers, 27 reported physical consequences, 25 revealed an impact on emotional well-being, 47 highlighted consequences related to work functioning, 10 showed a negative impact on the quality of the relationship and care with patients, and four studies reported financial and social consequences.

Several studies in the healthcare sector have established a positive association between workplace violence and psychological distress [8,9,10,11], and between workplace violence and a lesser level of confidence in one’s own abilities to cope with patient aggression [12,13]. Martin and Daffern [14] highlighted the importance for mental healthcare workers, who are at high-risk of violence, to have confidence in their capacities to work with aggressive patients. Additionally, the perceived severity of aggressive behaviors may decrease following training, which could be a sign of an increased confidence to manage these events [3].

### 1.1. Prevention through Education and Training Programs

Within the literature, it is frequently recommended that employers implement education and training programs for high-risk workers in order to prevent workplace violence [3,5,6,15]. These programs generally aim to help workers develop skills to better recognize and react to violent situations, and to better cope with their consequences. They “comprise any of a broad range of techniques to enhance knowledge and understanding of organisational policies and procedures, legal responsibilities, and risk assessment and control strategies” [6]. Wang et al. [5] reviewed 35 studies conducted among nurses that assessed the effectiveness of workplace violence education and training programs. Their results showed that the majority of these programs decreased violence, improved knowledge and confidence, and increased tolerance and positive attitude change. They also identified several programs that had no influence on violence or that increased violence. In addition, a review of literature specific to acute hospital settings was conducted by Heckemann et al. [16] and showed that all nine education and training programs reviewed lead to increased confidence as well as improved attitude, skills, and knowledge. However, these authors noted no significant change in the incidence of violent acts in the workplace. Based on 38 studies conducted in mental health settings, Price, Baker, Bee, and Lovell [17] found that the greatest benefits of education and training programs appear to be de-escalation-related knowledge, confidence in managing aggression, and de-escalation performance. However, they could not draw conclusions about the impact of training on assaults, injuries, containment, and organizational outcomes, owing to the low quality of evidence and conflicting results.

The bulk of the literature on education and training programs for the prevention of workplace violence is limited by the short duration of evaluation periods. Among the cited reviews [5,16,17,18], the majority of the studies did not provide information on the duration of the evaluation period, and only four were based on a follow-up period of one year or more. Therefore, literature regarding intermediate and long-term effects of these programs is scarce.

### 1.2. Effectiveness of Education and Training Programs for Preventing Violence in the Healthcare Sector

Studies on education and training programs aimed at preventing workplace violence towards employees generally conclude that these are effective. However, measures are generally obtained immediately following the training or after a very short time period [5,16,18]. The current study investigates the effectiveness of the *Omega* training program in both a short-term and follow-up period. To date, only one study has evaluated the impact of this program, and shows that participants increased their knowledge on how to better prevent workplace violence [19]. No empirical studies have yet been conducted to assess the impact of the training program on the psychological health of participants or on the perceived risk of violence. Given that this program is widely used in Canada, it is necessary to assess its efficiency based on scientific facts and to develop recommendations.

### 1.3. The Omega Education and Training Program

The *Omega* program aims to prevent and minimize workplace aggression directed toward healthcare workers by improving the knowledge, attitudes, and skills of participants when facing verbal and physical aggression by patients. It was created in 1999 by the Health and Social Services section of the Agency for Health and Safety at Work of the province of Quebec, Canada [20]. Three counselors from the governmental Agency for Health and Safety at Work of the province of Quebec, as well as six workers from a mental health institute specialized in violence issues, designed the program over a two-year period. During this period, observational analyses and interviews were conducted in three psychiatric hospitals in order to identify the expertise of experienced security workers in solving at-risk and violent situations. Moreover, at the time the program was developed (i.e., 1996), no specific education and training program dedicated to psychiatric hospitals was available in the province of Quebec. Since its creation, the program has been implemented in numerous hospitals, local community service centres, youth protection centres, and community organizations [21]. Between 1999 and 2015, the *Omega* program was implemented amongst 47,408 workers in Canada (data provided by the governmental organization responsible for the program).

The *Omega* program is taught by peer trainers (security agents). It lasts four days, and seeks to teach participants the skills and intervention methods necessary to ensure their safety and that of their patients in situations of aggression [20]. On the first day, participants are taught the fundamental values and principles of *Omega*. The four core values are respect, professionalism, accountability, and security. The five principles are to protect oneself, to assess the situation, to predict behavior, to take the time, and to focus on the person. The second day focuses on a pacification approach and on a grid for classifying behaviors and levels of dangerousness of potentially aggressive individuals. The third day addresses the utilized tools for the intervention pyramid (i.e., behaviors to adopt according to the behavior). The last day is focused on post-incident reports and on feedback regarding the three other days. Practical exercises are conducted every day. For each of the principles, the training program teaches specific verbal, psychological, or physical intervention techniques for frequently-encountered situations. Levels of dangerousness include emotional tension, conditional cooperation, refractory behavior, destructive behavior, psychological intimidation, active resistance, physical aggression, serious assaults, and exceptional threats. Teamwork in facing situations of violence is also highly valued and integrated in the training. Participants are taught to resolve aggressive crisis situations with an approach that is centered on the experiences of the individual in question. Seven levels of interventions are classified in an intervention pyramid ranging from mitigation (i.e., resolving the aggressive crisis situation or the acute crisis with an approach focused on the safety of the patient) to physical intervention (i.e., last resort intervention, used when the patient or client has imposed an act of protection or control).

The training also provides the necessary tools to complete a post-incident report. This report is designed to assess the relevance and effectiveness of crisis management interventions after-the-fact through team feedback and monitoring.

### 1.4. The Present Study

The purpose of this study was to examine the effect of the *Omega* education and training program, after the training and at a follow-up, on employee psychological well-being, and more precisely on their psychological distress, on their confidence in their skills, and on their perceived level of exposure to different forms of violence.

## 2. Methods

This study was approved by the Ethics committee of the Institut Universitaire en Santé Mentale de Montréal (IUSMM; Montreal Mental Health University Institute, Montreal, QC, Cananda).

### 2.1. Participants

Between January and October 2012, 105 employees of the high-risk units of the IUSMM (i.e., intensive care, emergency department, and security) were offered to participate in a training program to better prevent and manage situations of aggression in the workplace. The IUSMM is one of the two major institutes dedicated to mental health in the city of Montreal. The total number of employees in the IUSMM is 1984, 105 of which worked in the high-risk units. The total number of admissions is about 1880 each year. In 2012, 156 acts of violence were officially reported to the employer. At the time of the study, there was no other education and training program offered.

On the first day of the program, the employees were invited to participate in the current study. They were informed of the longitudinal design of the study (with three measurement times), the confidential aspect of the research and the monetary compensation of $25 for each completed assessment. Eighty-nine of the 105 solicited employees participated at the first time of measurement, which indicates a response rate of 85% at Time 0. Of these, 80 and 63 participants completed the short-term (Time 1) and follow-up (Time 2) posttest questionnaires, which represents retention rates of 85% and 70.9%, respectively. For Time 1 and Time 2, participants were mailed the questionnaire via the internal mail system 90 days and 420 days after their training. Participants were invited to return the envelopes within two weeks. Questionnaires were received 109 days and 441 days on average after the training. A follow-up letter was mailed to reach those who had not yet forwarded their response two weeks following each measurement time. Participants were informed that they could complete the questionnaire during their working hours. Motives for documented dropouts were as follows: non-defined personal reasons (eight cases), absence for vacations (three cases), disease (two cases), and sick leave (one case).

### 2.2. Study Instruments

A questionnaire package was created for the purpose of this study. Instruments were standardized scales (i.e., psychological distress, confidence in coping) or were developed specifically for the present study (i.e., level of exposure to different forms of violence). The questionnaire was administered in paper-and-pencil form.

#### 2.2.1. General sociodemographics

The questionnaire package included items about general demographics including sex, age, marital status, work unit, work shift, and employment type and status.

#### 2.2.2. Psychological distress

Psychological distress was measured by the K6 scale developed by Kessler et al. [22]. Psychological distress was assessed based on the frequency with which participants had experienced anxiety and depression symptoms during the last month. The instrument consists of six items rated on a five-point scale ranging from “never” to “all the time”. A higher score is indicative of greater psychological distress. The Cronbach alpha was 0.76 in this study. A score of 13 or more indicates a risk for severe psychological distress.

#### 2.2.3. Level of exposure to different forms of violence

Three scales were designed to evaluate perceived exposure to tensions, minor violence, and serious violence, respectively, during the last three months. These items referred specifically to the definition of various levels of violence defined in the *Omega* training. Participants were told: “This section is about your personal experience of specific events and their frequency in the intensive care and emergency units. For each of the following statements, please assess the frequency with which these events occurred during the last 3 months.” Each scale consists of three items, each of which was assessed on a six-point scale from “never” to “everyday”. An example item for tensions is: “A patient is anxious, crying or has isolated himself/herself”; for minor violence: “A patient throws or breaks objects”; for serious violence: “A patient commits acts that might cause injury or death”. The Cronbach alphas were 0.85, 0.78, and 0.77. No standardized measures were available.

#### 2.2.4. Confidence in coping with patient aggression

Confidence in coping with patient aggression was measured with the scale developed by Thackrey [23]. The instrument consists of 10 items rated on an 11-point scale. Ranges vary depending the question from “very uncomfortable”, “very poor”, “very unable”, “very unsafe”, and “very ineffective” to “very comfortable”, “very good”, “very able”, “very safe”, and “very effective”. A high score is indicative of strong confidence in dealing with patient aggression. The Cronbach alpha was 0.96 in this study. This scale was found to be a useful instrument for evaluations on the group level when used as a pre- and post-test measure [24]. No standardized measures were available.

### 2.3. Analysis

Data analysis was performed using the Statistical Package for the Social Sciences (IBM SPSS Statistics for Windows, Version 19.0. Armonk, NY, USA) and using an alpha of 0.05. Repeated-measures ANOVAs were performed to estimate the differences in participants’ scores between the pretests and the two posttests. We conducted bivariate analysis and checked for potential confounding variables (sex, age, unit of work, type of employment, or work shift), for which only statistical significant changes are presented. Finally, Cohen’s *d* effect sizes were calculated after controlling for correlations between measurement times to determine the extent of the significant results. Criteria for Cohen’s *d* effect sizes proposed by Kotrlik, Williams, and Jabor [25] were used and were as followed: small (*d* ≥ 0.10), medium (*d* ≥ 0.30), and large (*d* ≥ 0.50).

## 3. Results

### 3.1. Demographic Variables

Participants’ sociodemographic characteristics at Time 0 are presented in Table 1.

Eighty-nine employees participated in the first measurement time of the study. A majority of them were 46 years old and over (56%) and had accumulated 10 or more years of experience (74.2%). Sociodemographic characteristics of participants that did not complete the surveys at Time 1 and Time 2 did not differ from the those of the participants that completed the three times measurement.

### 3.2. Measured Changes

At Time 1 and Time 2, almost all dependent variables showed significant changes from the baseline results (Table 2).

The perceived level of exposure to the different forms of violence under study diminished significantly across all times of measurement with the exception of exposure to tensions, the lesser level of violence, which decreased only for the T0–T1 period with a medium effect size. None of the participants obtained a score of 13 and more, which is the cut-off point for severe psychological distress. The means of exposure to minor violence significantly decreased across all measurement times with medium and large effect sizes. Exposure to minor violence varied according to age, with exposure to minor violence decreasing more among participants aged 45 years and older than for younger respondents between Time 1 and Time 2 (MD = −1.7 vs. −0.47, F(1,69) = 4.59, *p* = 0.036) and between Time 1 and Time 3 (MD = −0.63 vs. −0.77, F(1,55) = 9.56, *p* = 0.003). Exposure to serious violence also significantly declined across all measurement times with medium and large effect sizes. Exposure to serious violence varied according to sex, with men being more exposed to serious violence than women, regardless of measurement time (m = 4.32 vs. m = 2.89 for Time 1, t = 2.35, *p* = 0.021; m = 3.87 vs. m = 2.05, t = 3.51, *p* = 0.001 for Time 2; m = 2.58 vs. m = 1.72, t = 2.19, *p* = 0.033 for Time 3). Psychological distress in the last month decreased significantly for both comparisons with medium effect sizes. None of our participants reached or exceeded this score. Confidence in coping with patient aggression decreased significantly across time with medium and large effect sizes.

## 4. Discussion

The present study aimed to evaluate the efficacy of the *Omega* training program among mental health workers. This training program seeks to develop specific interpersonal skills and behavior management techniques to effectively intervene in situations of aggression, while ensuring the safety of participants and that of their patients.

Results showed significant improvements for all of the variables under study. This study suggests that the *Omega* program led to significant improvements in both the short-term and, to the exception of exposure to tensions, at follow-up, suggesting that the observed impact of the program is maintained over time. Moreover, a majority of the calculated effects sizes were medium or large, supporting the organizational relevance of the intervention. It is noteworthy that the effects remained strong despite our relatively small sample size.

The institution decided to move forward with the *Omega* training, offering it to all workers. The institution also participated in the dissemination of the results of the present study to all its workers (e.g., financial support for the writing of a report to workers, advertisement on the intranet and in the units for a series of lunch conferences about the results).

The positive impact of the *Omega* training program may be explained by the fact that this program meets the recommendations formulated in the literature regarding staff training to prevent workplace violence [26,27]. For instance, in their review of the literature on this subject, Beech and Leather [27] reported that a good training program should contain theory (understanding aggression and violence in the workplace), prevention (assessing danger and taking precautions), interaction (with aggressive people), and post-incident action (reporting, investigation, counselling, and other follow-up). The *Omega* training also includes recommendations made by Abu Al Rub et al. [26] according to whom a training program should include the recognition of verbal and nonverbal signs of aggression, risk assessment and management, de-escalation tactics, and post-incident support.

Our results on confidence in coping are congruent with the review of the literature of Price et al. [17], reporting that nine of ten studies showed significantly increased confidence post-training. To our knowledge, this is the first study demonstrating a decreased perceived level of exposure to violence. So far, literature has indicated that the impact on incident rates was mitigated, but findings were based on official data [17].

### 4.1. Limitations

Results need to be interpreted carefully due to several methodological issues. First, the design did not include a control group and, thus, external factors, such as organizational changes, may have accounted for the observed variations. However, we did not observe any major structural or administrative changes during the program evaluation. Moreover, having a control group amongst workers of the same units would have led to a high risk of contamination. Indeed, workers in a potential control group would have received a lot of information regarding the intervention while working with workers in the intervention group. For ethical reasons, it would have been impossible to forbid it. Moreover, it would have been difficult to create a control group prior to the implementation of the intervention due to the lengthy follow-up times. Finally, the units under study have specificities (high-risk behaviors, patients in severe distress or in decompensation) that are their own and, thus, no other units in the institution presented the same characteristics to serve as a control group. With regards to our measures in this study, we only focused on change in employees and did not measure perceived changes in patients. Additionally, because self-reporting measures are used, desirability can be a factor of change, as is true for all studies dealing with self-reporting measures. However, participation was voluntary and respondents were informed numerous times of the confidentiality of the study. Moreover, the research team was completely independent from the administrative team and management. These entities were only aware of participation for technical reasons (i.e., absence to complete the questionnaire). In addition, the attrition rate (29.2%) could have influenced the results. Indeed, attrition has statistical consequences [28]. However, rates of 50%–80% at follow-up are considered to be acceptable [28]. Moreover, our sample size was limited and did not allow us to conduct multivariate analyses, which would have been particularly useful to more accurately assess the impact of sociodemographic variables on the results.

### 4.2. Further Research

Additional research is required to evaluate the impact of training programs on objective measures, such as the number of violent incidents, given that changes in objective and subjective measures are not systematically correlated [5]. Further studies need to focus on the impact of the program on patient behavior. These measures could be based on official data (number, duration, and justification of isolation or contention) or on subjective data (assessment of the quality of the provider-patient relationship). Additional research should also consider larger samples to better assess the impact of sociodemographic characteristics through multivariate analyses and better identify resistant participants. This would allow researchers to further investigate the reasons for the absence of change among some participants and to better adapt training content. Research should also focus on solutions to increase the impact of the training program, for instance with reminders of the key concepts at a to-be determined time period via different sources of communication (email, text messages, awareness posters in organizations). Further research should consider the use and the impact of post-incident action on psychological well-being. Finally, longer measurement periods should be encouraged.

### 4.3. Clinical Implications

The training program was found to have a positive and significant impact on psychological distress, perception of security, and level of exposure to violent acts among employees in a mental health hospital. Since findings revealed positive outcomes, they bolster the dissemination of the *Omega* training program in similar clinical settings. A decrease in psychological distress could have a positive impact on the general wellbeing of workers. A decrease in the level of exposure to violent acts may positively affect the ways in which workers perceive their job, since studies have demonstrated that workplace violence is associated with loss of commitment, motivation, and risk of quitting [29,30]. The quality of the provider-patient relationship may also be enhanced.

## 5. Conclusions

In conclusion, this study demonstrates the positive impact of the *Omega* training program, which has been used in Canada since 1999 but had never been scientifically evaluated prior to this study. The majority of these effects remained consistent more than fourteen months following the completion of training.

## Figures and Tables

**Table 1:** Participants sociodemographic characteristics at Time 0 (*n* = 89).

Variables	*n* (%)
Sex	
Women	40 (45)
Men	49 (55)
Age	
≤35	17 (19.1)
36 to 45	22 (24.7)
≥46	50 (56.2)
Years of experience	
≤9	23 (25.8)
10 to 21	22 (24.7)
≥22	44 (49.5)
Employment status	
Permanent	50 (56.2)
Temporary	39 (43.8)
Work unit	
Emergency	41 (46.1)
Intensive care	26 (29.2)
Security	22 (24.7)
Job title	
Nurses	35 (39.8)
Orderlies	25 (28.4)
Security agents	21 (23.9)
Other	7 (8.0)
Shift	
Day	37 (42.0)
Evening	29 (33.0)
Night	17 (19.3)
Other	5 (5.7)

**Table 2:** Within-subjects ANOVAS, contrasts, and effects size.

Comparisons	MD ^1^	DF ^2^	F	*d*	Sig
Exposure to tensions		2	4.15		0.018
Time 0–Time 1	−1.01	1	7.41	0.34	0.009
Time 0–Time 2	−1.15	1	1.68	-	0.202
Exposure to minor violence		2	10.17		0.0001
Time 0–Time 1	−1.11	1	12.08	0.39	0.001
Time 0–Time 2	−1.60	1	21.63	0.64	0.0001
Exposure to violent acts		2	8.92		0.0001
Time 0–Time 1	−0.58	1	5.26	0.34	0.026
Time 0–Time 2	−1.20	1	13.46	0.63	0.001
Psychological distress		2	8.67		0.0001
Time 0–Time 1	−1.00	1	10.71	0.33	0.002
Time 0–Time 2	−1.10	1	12.96	0.30	0.001
Confidence in coping scale		2	13.50		0.0001
Time 0–Time 1	7.87	1	11.48	−0.44	0.001
Time 0–Time 2	8.34	1	24.99	−0.64	0.0001

Note: ^1^ MD: mean differences; ^2^ DF: degrees of freedom.
